# Special Issue “Cytokines in Inflammatory Signaling: 2nd Edition”

**DOI:** 10.3390/ijms27042056

**Published:** 2026-02-22

**Authors:** Wai Po Chong

**Affiliations:** 1 School of Chinese Medicine, Hong Kong Baptist University, Hong Kong 999077, China; chongwp@hkbu.edu.hk; Tel.: +852-3411-2928; 2 Institute for Research and Continuing Education, Hong Kong Baptist University, Shenzhen 518000, China

Cytokines serve as critical mediators of the immune response, orchestrating host defense against pathogens, cellular proliferation, and tissue homeostasis; however, dysregulation of cytokine signaling can precipitate chronic inflammation, autoimmune disorders, and metabolic diseases, underscoring the delicate balance required for physiological integrity. This Special Issue, “Cytokines in Inflammatory Signaling: 2nd Edition”, delves into the molecular mechanisms by which cytokines influence inflammatory pathways across diverse contexts, ranging from gut health to neurodegeneration. The collection comprises nine original research articles and one comprehensive review, which together advance our understanding of cytokine networks and their therapeutic implications. 

**Gut Inflammation and the Microbiome**

The interplay between cytokines, gut microbiota, and intestinal immunity emerges as a pivotal theme. Ahmed et al. explore the probiotic potential of *Lactobacillus murinus* in mitigating TNF-α-induced inflammation in human intestinal epithelial cells (contribution 1). Their work reveals that *L. murinus* activates the aryl hydrocarbon receptor (AHR), upregulating CYP1A1 expression and attenuating epithelial barrier disruption and IL-8 secretion. This suggests AHR signaling as a mechanistic basis for probiotic efficacy, though the specific bacterial metabolites involved remain to be identified. Complementing this, Nualart et al. investigate the immunomodulatory impact of the antibiotic oxytetracycline (OTC) in coho salmon (contribution 2). Their in vivo and in vitro analyses demonstrate that OTC provokes a pro-inflammatory response in intestinal tissues, marked by upregulation of TLRs, NF-κB, and IL-6. These findings highlight the unintended consequences of antibiotic use in aquaculture and emphasize the need for optimized dosing to preserve immune homeostasis. 

**Wound Healing and Tissue Repair**

Cytokine dynamics in tissue regeneration are elucidated through non-mammalian models. Kuciński et al. characterize full-thickness skin wound healing in maraena whitefish, documenting the conserved stages of repair—re-epithelialization, inflammation, and remodeling—accompanied by temporally regulated expression of immune genes such as IL-17D and TGF-β (contribution 3). This study acts not only as a detailed histological atlas but also identifies cytokine-driven mechanisms shared across vertebrates, offering insights for improving piscine health in aquaculture and informing regenerative medicine. 

**Obesity, Metabolic Health, and Adipose Tissue Dysfunction**

Adipose tissue inflammation and metabolic dysregulation are dissected across multiple studies. Al-Dallal et al. challenge prevailing assumptions by examining resistin in Mexican-Americans, revealing no significant link between resistin levels and obesity or metabolic health—a contrast to studies in other ethnic groups (contribution 4), underscoring the importance of ethnic-specific factors in cytokine biology. Ostrowska et al. further probe adipose dysfunction, proposing IL-6 and resistin as gender-specific markers of obesity-related inflammation, with women exhibiting elevated resistin and MMP-2 (contribution 5). Expanding on cellular mechanisms, Chen et al. demonstrate that saturated fatty acids polarize skeletal muscle-resident macrophages toward a pro-inflammatory state, while unsaturated fatty acids modulate anti-inflammatory responses in adipose tissue (contribution 6). This niche specificity highlights the complexity of immunometabolic crosstalk in obesity. 

**Neuroinflammation and Alzheimer’s Disease**

The role of cytokines in neurodegeneration is explored through experimental and review perspectives. Frago et al. demonstrate that glycine–proline–glutamate (GPE) co-administration reduces amyloid-β (Aβ) deposition in the hippocampus of a rat model, correlating with normalized IGF-1 signaling, somatostatin function, and cytokine balance (e.g., IL-2 and IL-13) (contribution 7). This positions GPE as a promising neuroprotective agent. Chen et al. (Zilin) provide a systematic review of cytokine networks in Alzheimer’s disease, detailing how pro-inflammatory actors (e.g., IL-1β and TNF-α) drive neurotoxicity, while anti-inflammatory cytokines (e.g., IL-33 and IL-35) may offer protection (contribution 8). They advocate for cytokine-targeted therapies to ameliorate neuroinflammation. 

**Autoimmunity and Macrophage Polarization**

Macrophage plasticity and cytokine signaling in autoimmunity are scrutinized in depth. Bae et al. link chronic low-level IFN-γ exposure to mitochondrial complex I dysfunction in renal macrophages, identifying this disruption as an early event in lupus nephritis pathogenesis (contribution 9) and implicating mitochondrial integrity as a therapeutic target. Yao et al. review macrophage polarization in antiviral immunity, describing how viruses exploit M1/M2 dynamics to evade host defenses (contribution 10). They frame polarization as a “double-edged sword,” balancing viral clearance against immunopathology, and propose macrophage-focused interventions for viral diseases. 

**Concluding Remarks**

The contributions to this Special Issue illuminate the multifaceted roles of cytokines in inflammation, spanning microbial interactions, metabolic dysregulation, tissue repair, neurodegeneration, and autoimmunity. Key advances include the identification of microbial and dietary modulators of cytokine networks, ethnic- and gender-specific immune profiles, mitochondrial dysfunction as a driver of autoimmunity, and conserved repair mechanisms across species. Collectively, these studies underscore the therapeutic potential of targeting cytokine signaling while advocating for personalized approaches that account for genetic, environmental, and physiological variables. Future research should prioritize elucidating causal mechanisms, identifying novel metabolites, and translating findings into clinical interventions. We extend our gratitude to the authors for their rigorous work and hope that this Issue catalyzes further innovation in this field.

## Data Availability

Not applicable.

